# Supplementary material for: Prevalence and risk factors of uncorrected refractive error among an elderly Chinese population in urban China: a cross-sectional study
Source: BMJ Open. 2018 Mar 19;8(3):bmjopen-2017-021325. doi: 10.1136/bmjopen-2017-021325 (PMC5875627; doi:10.1136/bmjopen-2017-021325)
Supplement: Author's manuscript [file bmjopen-2017-021325.draft_revisions.pdf]

BMJ Open is committed to open peer review. As part of this commitment we make the peer review history of every article we publish publicly available.

When an article is published we post the peer reviewers' comments and the authors' responses online. We also post the versions of the paper that were used during peer review. These are the versions that the peer review comments apply to.

The versions of the paper that follow are the versions that were submitted during the peer review process. They are not the versions of record or the final published versions. They should not be cited or distributed as the published version of this manuscript.

BMJ Open is an open access journal and the full, final, typeset and author-corrected version of record of the manuscript is available on our site with no access controls, subscription charges or pay-per-view fees (http://bmjopen.bmj.com).

If you have any questions on BMJ Open's open peer review process please email info.bmjopen@bmj.com

BMJ Open

Prevalence and Risk Factors of Uncorrected Refractive Error among an Elderly Chinese Population in Urban China: The Jiangning Eye Study

Journal:	*BMJ Open*
Manuscript ID	bmjopen-2017-021325
Article Type:	Research
Date Submitted by the Author:	21-Dec-2017

Complete List of Authors:	Ye, Hehua; Shanghai Jiao Tong University, Department of Ophthalmology, Xinhua Hospital<br>Qian, Yiyong; Shanghai Tenth People's Hospital, Tongji University School of Medicine, Department of Ophthalmology<br>Zhang, Qi; Shanghai Jiao Tong University, Department of Ophthalmology, Xinhua Hospital<br>Liu, Xiaohong; Shanghai Jiao Tong University Affiliated Sixth People's Hospital, Department of Ophthalmology<br>Cai, Xuan; Shanghai Jiao Tong University Affiliated Sixth People's Hospital, Department of Ophthalmology<br>Yu, Wenjing; Shanghai Jiao Tong University, Department of Ophthalmology, Xinhua Hospital<br>Li, Xiang; Shanghai Jiao Tong University, Department of Ophthalmology, Xinhua Hospital<br>Zhao, Peiquan; Shanghai Jiao Tong University, Department of Ophthalmology, Xinhua Hospital

Keywords:	refractive error, prevalence, risk factor, epidemiology

SCHOLARONE™
Manuscripts

Title Page

Prevalence and Risk Factors of Uncorrected Refractive Error among an Elderly Chinese Population in Urban China: The Jiangning Eye Study

Hehua Ye,[1*] Yiyong Qian,[2*] Qi Zhang,[1] Xiaohong Liu,[3] Xuan Cai,[3] Wenjing Yu,[1] Xiang Li,[1] Peiquan Zhao[1]

[1]Department of Ophthalmology, Xinhua Hospital, Shanghai Jiao Tong University School of Medicine, Shanghai, China

[2]Department of Ophthalmology, Shanghai Tenth People's Hospital, Tongji University School of Medicine, Shanghai, China

[3]Department of Ophthalmology, Shanghai Jiao Tong University Affiliated Sixth People's Hospital, Shanghai, China

*HY and YQ contributed equally to this study.

Correspondence to:

Peiquan Zhao, MD, PhD

Department of Ophthalmology

Xinhua Hospital, Shanghai Jiao Tong University School of Medicine

1665 Kongjiang Rd, Shanghai, 200092 P.R. China

Phone: 86-21-25078505

Fax: 86-21- 65011178

E-mail: zhaopeiquan@126.com; zhaopeiquan@xinhuamed.com.cn

For peer review only

For peer review only - http://bmjopen.bmj.com/site/about/guidelines.xhtml

ABSTRACT

Objectives: To investigate the prevalence and risk factors of uncorrected refractive error (URE) in an elderly urban Chinese population in China.

Design: A population-based cross-sectional study.

Methods: The study was conducted using a cluster random sample of residents aged 50 years or older living in the Jiangning Road sub-district, Shanghai, China. All participants underwent a standardized interview and comprehensive eye examinations, including presenting visual acuity (PVA) and best-corrected visual acuity (BCVA) between November 2012 and February 2013. URE was defined as an improvement of two lines or more in the BCVA compared with the PVA in the better eye of < 20/40.

Results: A total of 1,999 subjects (an 82.5% response rate) completed both the questionnaire and ophthalmic examination. The prevalence of URE was 20.1% (95% confidence interval [CI] = 18.0%-22.2%) in the study sample. After age standardization, the prevalence of URE in Chinese people aged 50 years or older was 18.7% (95% CI = 17.0%-20.4%). Under multiple logistic regression analysis, older age (per 1-year increase, odds ratio [OR] = 1.04, 95% CI = 1.03-1.05) and a lower level of education (OR = 1.34, 95% CI = 1.07-1.69) were significantly related to URE. A history of ocular diseases (OR = 0.71, 95% CI = 0.55-0.92) was a protective factor for URE.

Conclusions: URE is highly prevalent among the elderly urban Chinese population, which should raise awareness of the URE burden in China to meet

For peer review only - http://bmjopen.bmj.com/site/about/guidelines.xhtml

the Vision 2020 goal to eliminate preventable blindness.

Strengths and limitations of this study

- A high response rate in a large population-based sample.
- Standardized protocols based on the typical definition of uncorrected refractive error.
- Uncorrected of near vision, that is, presbyopia, has not been evaluated in this population.
- The underlying reasons of uncorrected refractive error highly prevalent among elderly urban Chinese remains unknown.

INTRODUCTION

Uncorrected refractive error (URE) is the most common cause of vision impairment and the secondary cause of blindness worldwide. It has been estimated that URE accounts for 153 million individuals of visual impairment globally, and the World Health Organization (WHO) identified URE as one of the priorities for the program of "VISION 2020".[1]

URE is associated with limitations in vision-related tasks and decreased quality of life.[2 3] Despite the relatively easy intervention for refractive error, many people still suffer from vision impairment due to URE, especially older persons. Improvement in the vision-dependent quality of life of older persons has been demonstrated when URE is corrected.[4 5]

For peer review only - http://bmjopen.bmj.com/site/about/guidelines.xhtml

A wide variation in the prevalence of URE worldwide has been reported.[6] In East Asian countries, the prevalence of URE is potentially higher due to a higher prevalence of refractive errors.[7] China comprises one-fifth of the world's population with 78 million aged 60 years and above, and a substantial increase in the number of older persons is expected in the next few decades.[8] Despite the potential magnitude of this problem, there have been few population-based studies on URE in older persons in China.[9 10]

The purpose of the present study was to describe the prevalence and risk factors of URE in an elderly population in Shanghai, which is the largest city by population in China. The findings of this study may be helpful in determine the strategy to meet the Vision 2020 goal to eliminate preventable blindness.

METHODS

Study population

The Jiangning Eye Study, a population-based cross-sectional study of urban Chinese elders aged 50 years and older living in the Jiangning Road, Jing'an District, Shanghai, was conducted to assess the prevalence and risk factors of ocular diseases. The study design and details of population sampling have been described elsewhere.[11 12] The study followed the guidelines in the Declaration of Helsinki, and was approval by the Medical Ethics Committee of the Xinhua Hospital Affiliated to Shanghai Jiao Tong University School of Medicine. Informed written consent was obtained from each participant.

For peer review only - http://bmjopen.bmj.com/site/about/guidelines.xhtml

Study Procedures

An interviewer-administrated questionnaire was conducted to gather information about each participant's demographics, lifestyle (e.g., cigarette smoking and alcohol consumption), socioeconomic status factors (e.g., marital status, income level, and final education level), medical history, and history of ocular diseases. The ocular examination was conducted according to a standardized protocol included the presenting visual acuity (PVA) and best-corrected visual acuity (BCVA), autorefraction and subjective refraction, noncontact tonometry, slit-lamp biomicroscopy, and indirect ophthalmoscopy. Distance visual acuity was assessed using ETDRS charts and was recorded separately for each eye. All participants were asked to bring their spectacles before performing ocular examination. The PVA was measured with the subject's spectacles. If the participants did not wear spectacles or did not bring their spectacles, the PVA was measured without spectacles. If the PVA < 20/20, the BCVA was assessed with subjective refraction.

Definitions

URE was defined as an improvement of two lines (0.2 logMAR) or more in the BCVA compared with the PVA in the better eye of < 20/40.[13 14] Refractive status was expressed using the spherical equivalent (SE; sphere + 1/2 cylinder) calculated from the BCVA. An SE between -1.0 and +1.0 D was defined as emmetropia, an SE < -1.0 D as myopia, and an SE > +1.0 as hyperopia.

Statistical Analysis

For peer review only - http://bmjopen.bmj.com/site/about/guidelines.xhtml

The overall prevalence (%) of URE was calculated. The age standardized prevalence was calculated using direct standardization of the study samples to the 2010 Chinese population census.[8] A multiple logistic regression analysis was assessed with URE as the dependent variable. The relevant predictors were used as the covariates. Statistical analysis was performed with the Statistical Package for Social Science (SPSS 15.0, SPSS, Chicago, IL) software. A *P* value less than .05 was considered statistically significant.

RESULTS

Participants and descriptive data

Of 2,478 eligible participants identified for the Jiangning Eye Study, 2,044 (82.5% response rate) underwent ocular examinations in a temporary clinic. Data from 1,999 subjects completed both the questionnaire and ophthalmic examination were included and analyzed in the present study.

The mean age (± standard deviation) was 64.7 (± 9.9) years, and 56.2% were women. The age distribution of population was 50-59, 757 (37.9%); 60-69, 672 (33.6%); 70-79, 352 (17.6%), and 80 years or older, 218 (10.9%). The high correlation found between the right and left eyes of refractive status (r = 0.83; P < 0.001). Of the participants, 30.6% were myopic and 39.6% were hyperopic in the right eye.

The prevalence of URE

The prevalence rate of URE in the Jiangning Eye Study is summarized in

For peer review only - http://bmjopen.bmj.com/site/about/guidelines.xhtml

Table 1. The crude prevalence rate of URE in the entire study sample was 20.1% (95% confidence interval [CI], 18.0%-22.2%). After age standardization to the 2010 Chinese population census, the prevalence of URE in Chinese people aged 50 years or older was estimated to be 18.7% (95% CI, 17.0%-20.4%). There was a significant age-related trend in the prevalence of URE in the entire study sample (*P* value for the trend was < 0.001). The prevalence rate of URE in women (20.7%) was slightly higher than that in men (19.3%) (X^2 = 0.613, *P* = 0.434). Among those who wore spectacles or contact lenses (only 2 participants wore contact lenses), 16.5% we still uncorrected (i.e., a gain of 2 or more lines).

Analysis of associated factors

Table 2 summarizes the age- and multivariate-adjusted logistic regression model of the predictors for URE. URE was significantly associated with older age (per 1-year increase, odds ratio [OR] = 1.05, 95% CI = 1.04-1.06). After adjusting for age, a lower level of education (OR = 1.36, 95% CI = 1.09-1.70) was a significant risk factor for URE. On the other hand, a history of ocular disease (OR = 0.71, 95% CI = 0.55-0.91) was a significant protective factor for URE. In the final multiple logistic regression analysis, older age (per 1-year increase, OR = 1.04, 95% CI = 1.03-1.05) and a lower level of education (OR = 1.34, 95% CI = 1.07-1.69) were still significantly associated with URE, whereas a history of ocular disease (OR = 0.71, 95% CI = 0.55-0.92) was negatively associated with URE.

For peer review only - http://bmjopen.bmj.com/site/about/guidelines.xhtml

DISCUSSION

Key results

This population-based study provides novel data on the prevalence of URE in an elderly urban Chinese population in China. URE was present in 20.1% of the study sample, defining URE as an improvement of two lines (0.2 logMAR) or more in the BCVA compared with the PVA in the better eye of < 20/40. Older age and a lower level of education were significantly related to URE, whereas a history of ocular diseases was a protective factor.

Prevalence of URE

Visual impairment in elderly is of increasing importance with longer life expectancy and the resultant growing senior population.[15] Refractive error is the most common cause of vision impairment when people age.[16] Since Schwab and Tielsch drew attention to the importance of correctable vision impairment, URE has gained increasing attention in recent years as a major cause of avoidable blindness and visual impairment.[17 18]

Given that refractive error is more common in East Asia, a great number of individuals with URE would be expected among the older Chinese population.[7] However, a great disparity in the prevalence of URE in older Chinese has been presented in previous population-based studies (Table 3).[9 10 14 19 20] Our prevalence rate of URE is markedly higher than the rates in the Liwan Eye Study (7.0%; URE was defined as an improvement to 20/40 or better with

For peer review only - http://bmjopen.bmj.com/site/about/guidelines.xhtml

automated refraction),[9] the Shihpai Eye Study (9.6%; URE was defined as improving to better than 20/40 on refraction),[19] and the Hong Kong Study (13.4%; URE was defined as improving with pinhole to better than 20/60).[20] Although potential sources of errors exist in the recruitment ages and sampling methods, the discrepancies may be mainly due to differences in the definition of URE. Accordingly, the prevalence of URE in our study is similar to that in Singaporean-Chinese elders (21.7% in the Tanjong Pagar Study),[14] when the definition of URE is same. A previous study investigated in a rural block of Shanghai also showed a similar prevalence rate of URE (24.8% in the Baoshan Study) using a similar definition of URE.[10] Our finding expands the data suggesting that URE is a significant problem among older Chinese.

Factors associated with URE

In the risk factors analysis, older age has been shown to be significantly associated with increasing risk of URE. In this study, the prevalence rate of URE increased with age from 15.5% in the 50-59 range to 36.7% in participants older than 80 years. This age-related trend was in accordance with previous population-based studies.[13 14 19 21-23] In addition, people with a low level of education were associated with URE. This is probably because a lower educational level may result in a lower socioeconomic status and a lack of awareness of refractive errors.[13 14 19 21 23] On the other hand, people with a history of ocular diseases were negatively associated with URE, which may be due to more ophthalmic services accessed than those without a history of

For peer review only - http://bmjopen.bmj.com/site/about/guidelines.xhtml

ocular diseases. However, we could not confirm the previous association of URE with women.[13]

Novel insight into the public health strategy

Both URE and cataract have been included in the priority areas of the global initiative VISION 2020: The Right to Sight to eliminate preventable blindness.[6] With more than 20% of the world's population residing in China alone, great efforts have been made in China to meet the goal of Vision 2020. According to the National Plan for the Prevention and Treatment of Blindness, the coverage of cataract surgery has increased significantly through projects such as Free Cataract Surgeries for A Million Poor Patients in China.[24] The cataract surgery rate (CSR) in the Shanghai area increased from 1,741 in 2006 to 4,822 in 2016.[25 26] However, the findings of our study demonstrate the important contribution of URE to avoidable visual impairment in elderly Chinese aside from cataract and should raise awareness of the URE burden in China. An appropriate and cost�effective intervention for URE in older population should be considered during national health policy-making.

Strengths and limitations of the study

The strengths of the present study include a high response rate in a large population-based sample, and standardized protocols based on the typical definition of URE. However, this study has several limitations. First, refractive error that impairs near vision, that is, presbyopia, was not assessed. Second, the gross domestic product per capita in Shanghai has approached the level of

For peer review only - http://bmjopen.bmj.com/site/about/guidelines.xhtml

developed countries. All the subjects in the present study have health insurance, and ophthalmic consultations are easily accessed in this metropolitan area. We did not ask the reasons why they remained uncorrected for those subjects with URE in the present study, considering that economic factors and ophthalmic services should no longer be barriers. Further studies are needed to assess these issues.

Conclusions

In summary, this study suggests that URE is highly prevalent among the elderly Chinese population in urban China. These data provide a novel insight into the public health strategy for the Vision 2020 quest to prevent avoidable blindness and visual impairment in the world's most populous nation. Further investigation is needed to identify the effects of URE on the quality of life of the elderly and the underlying reasons why they remain uncorrected.

Acknowledgments The authors thanks Jian Zhang (Zhongshan Ophthalmic Center, Sun Yat-sen University, Guangzhou, China) for providing suggestions on study sampling and statistical analysis.

Contributors HY and PZ conceived and designed the study; HY, YQ, XLiu, XC, WY, and XLi performed the study; HY, YQ, and Q.Z. analyzed the data; HY, YQ, and PZ wrote the paper. All the authors read and approved the submitted version of the manuscript.

Competing interests None declared.

For peer review only - http://bmjopen.bmj.com/site/about/guidelines.xhtml

Ethics approval Medical Ethics Committee of the Xinhua Hospital Affiliated to Shanghai Jiao Tong University School of Medicine (XHEC-C-2012-014).

Data sharing statement No additional data are available.

REFERENCES

1. Resnikoff S, Pascolini D, Mariotti SP, et al. Global magnitude of visual impairment caused by uncorrected refractive errors in 2004. *Bull World Health Organ* 2008;86(1):63-70.

2. Smith TS, Frick KD, Holden BA, et al. Potential lost productivity resulting from the global burden of uncorrected refractive error. *Bull World Health Organ* 2009;87(6):431-7.

3. Dandona R, Dandona L. Refractive error blindness. *Bull World Health Organ* 2001;79(3):237-43.

4. Owsley C, McGwin G, Jr., Scilley K, et al. Effect of refractive error correction on health-related quality of life and depression in older nursing home residents. *Arch Ophthalmol* 2007;125(11):1471-7. doi: 10.1001/archopht.125.11.1471

5. Coleman AL, Yu F, Keeler E, et al. Treatment of uncorrected refractive error improves vision-specific quality of life. *J Am Geriatr Soc* 2006;54(6):883-90. doi: 10.1111/j.1532-5415.2006.00817.x

6. Schneider J, Leeder SR, Gopinath B, et al. Frequency, course, and impact of correctable visual impairment (uncorrected refractive error). *Surv*

For peer review only

Ophthalmol 2010;55(6):539-60. doi: 10.1016/j.survophthal.2010.02.004

7. McCarty CA. Uncorrected refractive error. *Br J Ophthalmol* 2006;90(5):521-2. doi: 10.1136/bjo.2006.090233

8. Tabulation on the 2010 Population Census of the People's Republic of China. Available at http://www.stats.gov.cn/english/Statisticaldata/CensusData/rkpc2010/indexch.htm. Accessed December 6, 2017.

9. Huang S, Zheng Y, Foster PJ, et al. Prevalence and causes of visual impairment in Chinese adults in urban southern China. *Arch Ophthalmol* 2009;127(10):1362-7. doi: 10.1001/archophthalmol.2009.138

10. Zhu M, Tong X, Zhao R, et al. Visual impairment and spectacle coverage rate in Baoshan district, China: population-based study. *BMC Public Health* 2013;13:311. doi: 10.1186/1471-2458-13-311

11. Ye H, Zhang Q, Liu X, et al. Prevalence and associations of epiretinal membrane in an elderly urban Chinese population in China: the Jiangning Eye Study. *Br J Ophthalmol* 2015;99(12):1594-7. doi: 10.1136/bjophthalmol-2015-307050

12. Ye H, Zhang Q, Liu X, et al. Prevalence of age-related macular degeneration in an elderly urban chinese population in China: the Jiangning Eye Study. *Invest Ophthalmol Vis Sci* 2014;55(10):6374-80. doi: 10.1167/iovs.14-14899

13. Rosman M, Wong TY, Tay WT, et al. Prevalence and risk factors of

For peer review only

For peer review only - http://bmjopen.bmj.com/site/about/guidelines.xhtml

undercorrected refractive errors among Singaporean Malay adults: the Singapore Malay Eye Study. *Invest Ophthalmol Vis Sci* 2009;50(8):3621-8. doi: 10.1167/iovs.08-2788

14. Saw SM, Foster PJ, Gazzard G, et al. Undercorrected refractive error in Singaporean Chinese adults: the Tanjong Pagar survey. *Ophthalmology* 2004;111(12):2168-74. doi: 10.1016/j.ophtha.2004.05.032

15. Olshansky SJ, Carnes BA, Cassel CK. The aging of the human species. *Sci Am* 1993;268(4):46-52.

16. Attebo K, Ivers RQ, Mitchell P. Refractive errors in an older population: the Blue Mountains Eye Study. *Ophthalmology* 1999;106(6):1066-72. doi: 10.1016/S0161-6420(99)90251-8

17. Tielsch JM, Sommer A, Witt K, et al. Blindness and visual impairment in an American urban population. The Baltimore Eye Survey. *Arch Ophthalmol* 1990;108(2):286-90.

18. Schwab L, Steinkuller PG. Visual disability and blindness secondary to refractive errors in Africa. *Soc Sci Med* 1983;17(22):1751-4.

19. Kuang TM, Tsai SY, Hsu WM, et al. Correctable visual impairment in an elderly Chinese population in Taiwan: the Shihpai Eye Study. *Invest Ophthalmol Vis Sci* 2007;48(3):1032-7. doi: 10.1167/iovs.06-0616

20. Michon JJ, Lau J, Chan WS, et al. Prevalence of visual impairment, blindness, and cataract surgery in the Hong Kong elderly. *Br J Ophthalmol* 2002;86(2):133-9.

For peer review only - http://bmjopen.bmj.com/site/about/guidelines.xhtml

21. Foran S, Rose K, Wang JJ, et al. Correctable visual impairment in an older population: the blue mountains eye study. *Am J Ophthalmol* 2002;134(5):712-9.

22. Dimitrov PN, Mukesh BN, McCarty CA, et al. Five-year incidence of bilateral cause-specific visual impairment in the Melbourne Visual Impairment Project. *Invest Ophthalmol Vis Sci* 2003;44(12):5075-81.

23. Varma R, Wang MY, Ying-Lai M, et al. The prevalence and risk indicators of uncorrected refractive error and unmet refractive need in Latinos: the Los AngelesLatino Eye Study. *Invest Ophthalmol Vis Sci* 2008;49(12):5264-73. doi: 10.1167/iovs.08-1814

24. Lin H, Lin D, Long E, et al. Patient participation in free cataract surgery: a cross-sectional study of the low-income elderly in urban China. *BMJ Open* 2016;6(4):e011061. doi: 10.1136/bmjopen-2016-011061

25. Zhu M, Zhu J, Lu L, et al. Four-year analysis of cataract surgery rates in Shanghai, China: a retrospective cross-sectional study. *BMC Ophthalmol* 2014;14:3. doi: 10.1186/1471-2415-14-3

26. THE LASTEST: Ranking of Chinese cataract surgery reporting situation in 2016. Available at: http://www.moheyes.com/(S(iwltcfbybpjl1x45sww14wjm))/News/Details/f5334a53-dd23-4bbe-a959-96c542b7262c. Accessed December 6, 2017.

For peer review only - http://bmjopen.bmj.com/site/about/guidelines.xhtml

Table 1. Prevalence rates of uncorrected refractive error by Gender and Age in the Jiangning Eye Study.

	All			Spectacle Wearers			Non-spectacle Wearers		
**Age Group (yrs.)**	***N***	**n**	**Prevalence Rate (%)**	***N***	**n**	**Prevalence Rate (%)**	***N***	**n**	**Prevalence Rate (%)**
Men									
50-59	297	35	11.8	81	8	9.9	216	27	12.5
60-69	321	47	14.6	120	14	11.7	201	33	16.4
70-79	158	46	29.1	48	11	22.9	110	35	31.8
80-95	99	41	41.4	28	7	25.0	71	34	47.9
Total population	875	169	19.3	277	40	14.4	598	129	21.6
Age-standardized prevalence (%)†			17.0 (14.5, 19.5)			13.3 (9.3, 17.3)			18.7 (15.6, 21.8)
P value for trend*			*P<0.001*			*P=0.013*			*P<0.001*
Women									
50-59	460	82	17.8	115	18	15.7	345	64	18.6
60-69	351	53	15.1	128	20	15.6	223	33	14.8
70-79	194	59	30.4	50	14	28.0	144	45	31.3
80-95	119	39	32.8	19	5	26.3	100	34	34.0
Total population	1124	233	20.7	312	57	18.3	812	176	21.7
Age-standardized prevalence (%)†			20.3 (17.9, 22.6)			18.5 (14.2, 22.9)			20.8 (18.0, 23.6)
P value for trend*			*P<0.001*			*P=0.079*			*P<0.001*
Both genders									
50-59	757	117	15.5	196	26	13.3	561	91	16.2
60-69	672	100	14.9	248	34	13.7	424	66	15.6
70-79	352	105	29.8	98	25	25.5	254	80	31.5
80-95	218	80	36.7	47	12	25.5	171	68	39.8
Total population	1999	402	20.1	589	97	16.5	1410	305	21.6
Age-standardized prevalence (%)†			18.7 (17.0, 20.4)			15.9 (13.0, 18.9)			19.7 (17.7, 21.8)
P value for trend*			*P<0.001*			*P=0.004*			*P<0.001*

**P* value for test of trend for age.

†Estimated prevalence (95% confidence interval) for projection by age-standardized to 2010 Chinese population census.

For peer review only

Table 2. Logistic Regression Model of the Predictors of Uncorrected Refractive Error.

Variable	*N*	n (%)	Age OR (95% CI) *	*P*	Multivariate OR (95% CI) †	*P*
**Age (per 1 year)**	1999	402 (20.1)	1.05 (1.04-1.06)	**<0.001**	1.04 (1.03-1.05)	**<0.001**
**Gender**				0.295		0.675
Male	875	169 (19.3)	1.0		1.0	
Female	1124	233 (20.7)	1.13 (0.90-1.41)		1.05 (0.84-1.32)	
**Education**				**0.008**		**0.019**
Secondary school and lower	902	217 (24.1)	1.36 (1.09-1.70)		1.34 (1.07-1.69)	
High school and above	1097	185 (16.9)	1.0		1.0	
**Income**				0.050		0.156
<5000	1327	292 (21.5)	1.28 (1.00-1.64)		1.20 (0.93-1.55)	
5000 and above	672	110 (16.4)	1.0		1.0	
**History of ocular diseases**				**0.007**		**0.009**
No	1500	315 (21.0)	1.0		1.0	
Yes	499	87 (17.4)	0.71 (0.55-0.91)		0.71 (0.55-0.92)	

OR = odds ratio; CI = confidence interval.

*Adjusted for age; †adjusted for age, gender, education, income, and history of ocular diseases.

For peer review only

For peer review only - http://bmjopen.bmj.com/site/about/guidelines.xhtml

Table 3. Prevalence rates and risk factors of uncorrected refractive error among Chinese population from the Jiangning and Other Eye Studies.

**Study (Year of Study)**	**Country/ Region**	***N***	**Age (y)**	**URE in Study Population (%)**	**Definitions (VA: visual acuity)**	**Multivariate Risk Factors**
Tanjong Pagar Survey[14] (1997-1998)	Singapore	1152	40–79	21.7%	BCVA - PVA ≥ 2 lines in the better eye of < 20/40	Older age, fewer years of education, not wearing spectacles, cataracts
Hong Kong Study[20] (1998)	Hong Kong	3441	60+	13.4%	Improving with pinhole to better than 20/60	NA
Shihpai Eye Study[19] (1999-2000)	Taiwan	1361	65+	9.6%	Improving to better than 20/40 on refraction	Older age, nonemmetropic eye, not wearing spectacles, lower level of education
Liwan Eye Study[9] (2003-2004)	China	1399	50+	7.0%	Improvement to 20/40 or better with automated refraction	NA
Baoshan Study[10] (2009)	China	4545	60+	24.8%	Improvement of two or more lines in VA in the better eye after refraction	NA
Jiangning Eye Study (2012-2013) (current)	China	1999	50+	20.1%	BCVA - PVA ≥ 2 lines in the better eye of < 20/40	Older age, secondary school and lower of education, no history of ocular diseases

BCVA = best-corrected visual acuity; PVA = presenting visual acuity; NA = not applicable

For peer review only

For peer review only - http://bmjopen.bmj.com/site/about/guidelines.xhtml

STROBE 2007 (v4) Statement—Checklist of items that should be included in reports of *cross-sectional studies*

**Section/Topic**	**Item #**	**Recommendation**	**Reported on page #**
**Title and abstract**	1	(*a*) Indicate the study's design with a commonly used term in the title or the abstract	Page 1, 2
		(*b*) Provide in the abstract an informative and balanced summary of what was done and what was found	Page 2
**Introduction**			
Background/rationale	2	Explain the scientific background and rationale for the investigation being reported	Page 3, 4
Objectives	3	State specific objectives, including any prespecified hypotheses	Page 4
**Methods**			
Study design	4	Present key elements of study design early in the paper	Page 4
Setting	5	Describe the setting, locations, and relevant dates, including periods of recruitment, exposure, follow-up, and data collection	Page 4
Participants	6	(*a*) Give the eligibility criteria, and the sources and methods of selection of participants	Page 4
Variables	7	Clearly define all outcomes, exposures, predictors, potential confounders, and effect modifiers. Give diagnostic criteria, if applicable	Page 4
Data sources/ measurement	8*	For each variable of interest, give sources of data and details of methods of assessment (measurement). Describe comparability of assessment methods if there is more than one group	Page 5
Bias	9	Describe any efforts to address potential sources of bias	*NA*
Study size	10	Explain how the study size was arrived at	Page 4
Quantitative variables	11	Explain how quantitative variables were handled in the analyses. If applicable, describe which groupings were chosen and why	NA
Statistical methods	12	(*a*) Describe all statistical methods, including those used to control for confounding	Page 6
		(*b*) Describe any methods used to examine subgroups and interactions	NA
		(*c*) Explain how missing data were addressed	NA
		(*d*) If applicable, describe analytical methods taking account of sampling strategy	Page 4
		(*e*) Describe any sensitivity analyses	Page 6
**Results**			

For peer review only

Participants	13*	(a) Report numbers of individuals at each stage of study—eg numbers potentially eligible, examined for eligibility, confirmed eligible, included in the study, completing follow-up, and analysed	Page 6
		(b) Give reasons for non-participation at each stage	NA
		(c) Consider use of a flow diagram	NA
Descriptive data	14*	(a) Give characteristics of study participants (eg demographic, clinical, social) and information on exposures and potential confounders	Page 6
		(b) Indicate number of participants with missing data for each variable of interest	Page 6
Outcome data	15*	Report numbers of outcome events or summary measures	Page 6
Main results	16	(*a*) Give unadjusted estimates and, if applicable, confounder-adjusted estimates and their precision (eg, 95% confidence interval). Make clear which confounders were adjusted for and why they were included	Page 7
		(*b*) Report category boundaries when continuous variables were categorized	Page 7
		(*c*) If relevant, consider translating estimates of relative risk into absolute risk for a meaningful time period	NA
Other analyses	17	Report other analyses done—eg analyses of subgroups and interactions, and sensitivity analyses	Page 7
**Discussion**			
Key results	18	Summarise key results with reference to study objectives	Page 8
Limitations	19	Discuss limitations of the study, taking into account sources of potential bias or imprecision. Discuss both direction and magnitude of any potential bias	Page 10, 11
Interpretation	20	Give a cautious overall interpretation of results considering objectives, limitations, multiplicity of analyses, results from similar studies, and other relevant evidence	Page 8-10
Generalisability	21	Discuss the generalisability (external validity) of the study results	Page 10
**Other information**			
Funding	22	Give the source of funding and the role of the funders for the present study and, if applicable, for the original study on which the present article is based	NA

*Give information separately for cases and controls in case-control studies and, if applicable, for exposed and unexposed groups in cohort and cross-sectional studies.

Note: An Explanation and Elaboration article discusses each checklist item and gives methodological background and published examples of transparent reporting. The STROBE checklist is best used in conjunction with this article (freely available on the Web sites of PLoS Medicine at http://www.plosmedicine.org/, Annals of Internal Medicine at http://www.annals.org/, and Epidemiology at http://www.epidem.com/). Information on the STROBE Initiative is available at www.strobe-statement.org.

For peer review only

BMJ Open

Prevalence and Risk Factors of Uncorrected Refractive Error among an Elderly Chinese Population in Urban China: a Cross-sectional Study

Journal:	*BMJ Open*
Manuscript ID	bmjopen-2017-021325.R1
Article Type:	Research
Date Submitted by the Author:	28-Jan-2018
Complete List of Authors:	Ye, Hehua; Shanghai Jiao Tong University, Department of Ophthalmology, Xinhua Hospital<br>Qian, Yiyong; Shanghai Tenth People's Hospital, Tongji University School of Medicine, Department of Ophthalmology<br>Zhang, Qi; Shanghai Jiao Tong University, Department of Ophthalmology, Xinhua Hospital<br>Liu, Xiaohong; Shanghai Jiao Tong University Affiliated Sixth People's Hospital, Department of Ophthalmology<br>Cai, Xuan; Shanghai Jiao Tong University Affiliated Sixth People's Hospital, Department of Ophthalmology<br>Yu, Wenjing; Shanghai Jiao Tong University, Department of Ophthalmology, Xinhua Hospital<br>Li, Xiang; Shanghai Jiao Tong University, Department of Ophthalmology, Xinhua Hospital<br>Zhao, Peiquan; Shanghai Jiao Tong University, Department of Ophthalmology, Xinhua Hospital
**Primary Subject Heading**:	Ophthalmology
Secondary Subject Heading:	Epidemiology
Keywords:	refractive error, prevalence, risk factor, Epidemiology < TROPICAL MEDICINE

SCHOLARONE™
Manuscripts

Title Page

Prevalence and Risk Factors of Uncorrected Refractive Error among an Elderly Chinese Population in Urban China: a Cross-sectional Study

Hehua Ye,[1*] Yiyong Qian,[2*] Qi Zhang,[1] Xiaohong Liu,[3] Xuan Cai,[3] Wenjing Yu,[1] Xiang Li,[1] Peiquan Zhao[1]

[1]Department of Ophthalmology, Xinhua Hospital, Shanghai Jiao Tong University School of Medicine, Shanghai, China

[2]Department of Ophthalmology, Shanghai Tenth People's Hospital, Tongji University School of Medicine, Shanghai, China

[3]Department of Ophthalmology, Shanghai Jiao Tong University Affiliated Sixth People's Hospital, Shanghai, China

*HY and YQ contributed equally to this study.

Correspondence to:

Peiquan Zhao, MD, PhD

Department of Ophthalmology

Xinhua Hospital, Shanghai Jiao Tong University School of Medicine

1665 Kongjiang Rd, Shanghai, 200092 P.R. China

Phone: 86-21-25078505

Fax: 86-21- 65011178

E-mail: zhaopeiquan@126.com; zhaopeiquan@xinhuamed.com.cn

For peer review only

For peer review only - http://bmjopen.bmj.com/site/about/guidelines.xhtml

ABSTRACT

Objectives: To investigate the prevalence and risk factors of uncorrected refractive error (URE) in an elderly urban Chinese population in China.

Design: A population-based cross-sectional study.

Methods: The study was conducted using a cluster random sample of residents aged 50 years or older living in the Jiangning Road sub-district, Shanghai, China. All participants underwent a standardized interview and eye examinations, including presenting visual acuity (PVA) and best-corrected visual acuity (BCVA) between November 2012 and February 2013. URE was defined as an improvement of two lines or more in the BCVA compared with the PVA in the better eye of < 20/40.

Results: A total of 1,999 subjects (an 82.5% response rate) completed both the questionnaire and ophthalmic examination. The prevalence of URE was 20.1% (95% confidence interval [CI] = 18.0%-22.2%) in the study sample. After age standardization, the prevalence of URE in Chinese people aged 50 years or older was 18.7% (95% CI = 17.0%-20.4%). Under multiple logistic regression analysis, older age (per 1-year increase, odds ratio [OR] = 1.04, 95% CI = 1.03-1.05) and a lower level of education (OR = 1.34, 95% CI = 1.07-1.69) were significantly related to URE. A history of ocular diseases (OR = 0.71, 95% CI = 0.55-0.92) was a protective factor for URE.

Conclusions: URE is highly prevalent among the elderly urban Chinese population, which should raise awareness of the URE burden in China to meet

For peer review only - http://bmjopen.bmj.com/site/about/guidelines.xhtml

the Vision 2020 goal to eliminate preventable blindness.

Strengths and limitations of this study

- A high response rate in a large population-based sample.
- Standardized protocols based on the typical definition of uncorrected refractive error.
- Uncorrected near vision, that is, presbyopia, has not been evaluated in this population.
- The underlying reasons of uncorrected refractive error highly prevalent among elderly urban Chinese remains unknown.

INTRODUCTION

Uncorrected refractive error (URE) is the most common cause of vision impairment and the secondary cause of blindness worldwide.[1] It has been estimated that URE accounts for 153 million individuals of visual impairment globally, and the World Health Organization (WHO) identified URE as one of the priorities for the program of ''VISION 2020''.[1]

URE is associated with limitations in vision-related tasks and decreased quality of life.[2 3] Despite the relatively easy intervention for refractive error, many people still suffer from vision impairment due to URE, especially older persons. Improvement in the vision-dependent quality of life of older persons has been demonstrated when URE is corrected.[4 5]

For peer review only

A wide variation in the prevalence of URE worldwide has been reported.[6] In East Asian countries, the prevalence of URE is potentially higher due to a higher prevalence of refractive errors.[7] Mainland China comprises one-fifth of the world's population with 78 million people aged 60 years and above, and a substantial increase in the number of older persons is expected in the next few decades.[8] Despite the potential magnitude of this problem, there have been few population-based studies on URE in older persons in mainland China.[9 10]

The purpose of the present study was to describe the prevalence and risk factors of URE in an elderly population in Shanghai, which is the largest city by population in China. The findings of this study may be helpful in determining the strategy to meet the Vision 2020 goal to eliminate preventable blindness.

METHODS

Study population

The Jiangning Eye Study, a population-based cross-sectional study of urban Chinese elders aged 50 years and older living in the Jiangning Road, Jing'an District, Shanghai, was conducted to assess the prevalence and risk factors of ocular diseases. The study design and details of population sampling have been described elsewhere.[11 12] The study followed the guidelines in the Declaration of Helsinki, and was approved by the Medical Ethics Committee of the Xinhua Hospital Affiliated to Shanghai Jiao Tong University School of Medicine. Informed written consent was obtained from each participant.

For peer review only - http://bmjopen.bmj.com/site/about/guidelines.xhtml

Study Procedures

An interviewer-administrated questionnaire was conducted to gather information about each participant's demographics, lifestyle (e.g., cigarette smoking and alcohol consumption), socioeconomic status factors (e.g., marital status, income level, and final education level), medical history, and history of ocular diseases. The ocular examination was conducted according to a standardized protocol included the presenting visual acuity (PVA) and best-corrected visual acuity (BCVA), autorefraction and subjective refraction, noncontact tonometry, slit-lamp biomicroscopy, and indirect ophthalmoscopy. Distance visual acuity was assessed using ETDRS charts and was recorded separately for each eye. All participants were asked to bring their spectacles before performing ocular examinations. The PVA was measured with the subject's spectacles. If the participants did not wear spectacles or did not bring their spectacles, the PVA was measured without spectacles. If the PVA < 20/20, the BCVA was assessed with subjective refraction.

Definitions

URE was defined as an improvement of two lines (0.2 logMAR) or more in the BCVA compared with the PVA in the better eye of < 20/40.[13 14] Refractive status was expressed using the spherical equivalent (SE; sphere + 1/2 cylinder) calculated from the BCVA. An SE between -1.0 and +1.0 D was defined as emmetropia, an SE < -1.0 D as myopia, and an SE > +1.0 as hyperopia.

Statistical Analysis

For peer review only - http://bmjopen.bmj.com/site/about/guidelines.xhtml

The overall prevalence (%) of URE was calculated. The age standardized prevalence was calculated using direct standardization of the study samples to the 2010 Chinese population census.[8] The correlation between the refractive status of the right and left eyes was calculated using the Spearman correlation coefficient. A multiple logistic regression analysis was assessed with URE as the dependent variable. The relevant predictors were used as the covariates. Statistical analysis was performed with the Statistical Package for Social Science (SPSS 15.0, SPSS, Chicago, IL) software. A P value less than .05 was considered statistically significant.

RESULTS

Participants and descriptive data

Of 2,478 eligible participants identified for the Jiangning Eye Study, 2,044 (82.5% response rate) underwent ocular examinations in a temporary clinic. Data from 1,999 subjects who completed both the questionnaire and ophthalmic examination were included and analyzed in the present study.

The mean age (± standard deviation) was 64.7 (± 9.9) years, and 56.2% were women. The age distribution of the population was 50-59, 757 (37.9%); 60-69, 672 (33.6%); 70-79, 352 (17.6%), and 80 years or older, 218 (10.9%). The high correlation found between the right and left eyes of refractive status (r = 0.83; $P < 0.001$). Of the participants, 30.6% were myopic and 39.6% were hyperopic in the right eye.

For peer review only - http://bmjopen.bmj.com/site/about/guidelines.xhtml

The prevalence of URE

The prevalence rate of URE in the Jiangning Eye Study is summarized in Table 1. The crude prevalence rate of URE in the entire study sample was 20.1% (95% confidence interval [CI], 18.0%-22.2%). After age standardization to the 2010 Chinese population census, the prevalence of URE in Chinese people aged 50 years or older was estimated to be 18.7% (95% CI, 17.0%-20.4%). There was a significant age-related trend in the prevalence of URE in the entire study sample (*P* value for the trend was < 0.001). No significant difference was found between men (19.3%) and women (20.7%) in the prevalence rate of URE (X^2 = 0.613, *P* = 0.434). Among those who wore spectacles or contact lenses (only 2 participants wore contact lenses), 16.5% were still uncorrected (i.e., a gain of 2 or more lines). Among the 101 participants with prior cataract surgery (at least one eye), 17.8% were uncorrected.

Analysis of associated factors

Table 2 summarizes the age- and multivariate-adjusted logistic regression model of the predictors for URE. URE was significantly associated with older age (per 1-year increase, odds ratio [OR] = 1.05, 95% CI = 1.04-1.06). After adjusting for age, a lower level of education (OR = 1.36, 95% CI = 1.09-1.70) was a significant risk factor for URE. On the other hand, a history of ocular disease (OR = 0.71, 95% CI = 0.55-0.91) was a significant protective factor for URE. In the final multiple logistic regression analysis, older age (per 1-year

For peer review only - http://bmjopen.bmj.com/site/about/guidelines.xhtml

increase, OR = 1.04, 95% CI = 1.03-1.05) and a lower level of education (OR = 1.34, 95% CI = 1.07-1.69) were still significantly associated with URE, whereas a history of ocular disease (OR = 0.71, 95% CI = 0.55-0.92) was negatively associated with URE.

DISCUSSION

Key results

This population-based study provides novel data on the prevalence of URE in an elderly urban Chinese population in China. URE was present in 20.1% of the study sample, defining URE as an improvement of two lines (0.2 logMAR) or more in the BCVA compared with the PVA in the better eye of < 20/40. Older age and a lower level of education were significantly related to URE, whereas a history of ocular diseases was a protective factor.

Prevalence of URE

Visual impairment in the elderly is of increasing importance with longer life expectancy and the resultant growing senior population.[15] Refractive error is the most common cause of vision impairment when people age.[16] Since Schwab and Tielsch drew attention to the importance of correctable vision impairment, URE has gained increasing attention in recent years as a major cause of avoidable blindness and visual impairment.[17 18]

Given that refractive error is more common in East Asia, a great number of individuals with URE would be expected among the older Chinese population.[7]

For peer review only - http://bmjopen.bmj.com/site/about/guidelines.xhtml

However, a great disparity in the prevalence of URE in older Chinese has been presented in previous population-based studies (Table 3).[9 10 14 19 20] Our prevalence rate of URE is markedly higher than the rates in the Liwan Eye Study (7.0%; URE was defined as an improvement to 20/40 or better with automated refraction),[9] the Shihpai Eye Study (9.6%; URE was defined as improving to better than 20/40 on refraction),[19] and the Hong Kong Study (13.4%; URE was defined as improving with pinhole to better than 20/60).[20] Although potential sources of errors exist in the recruitment ages and sampling methods, the discrepancies may be mainly due to differences in the definition of URE. Accordingly, the prevalence of URE in our study is similar to that in Singaporean-Chinese elders (21.7% in the Tanjong Pagar Study),[14] when the definition of URE is same. A previous study investigated in a rural block of Shanghai also showed a similar prevalence rate of URE (24.8% in the Baoshan Study) using a similar definition of URE.[10] Our finding expands the data suggesting that URE is a significant problem among older Chinese.

Factors associated with URE

In the risk factors analysis, older age has been shown to be significantly associated with increasing risk of URE. In this study, the prevalence rate of URE increased with age from 15.5% in the 50-59 range to 36.7% in participants older than 80 years. This age-related trend was in accordance with previous population-based studies.[13 14 19 21-23] In addition, people with a low level of education were associated with URE. This is probably because a lower

For peer review only - http://bmjopen.bmj.com/site/about/guidelines.xhtml

educational level may result in a lower socioeconomic status and a lack of awareness of refractive errors.[13 14 19 21 23] On the other hand, people with a history of ocular diseases were negatively associated with URE, which may be due to more ophthalmic services accessed than those without a history of ocular diseases. However, we could not confirm the previous association of URE with women.[13]

Novel insight into the public health strategy

Both URE and cataract have been included in the priority areas of the global initiative VISION 2020: The Right to Sight to eliminate preventable blindness.[6] With more than 20% of the world's population residing in China alone, great efforts have been made in China to meet the goal of Vision 2020. According to the National Plan for the Prevention and Treatment of Blindness, the coverage of cataract surgery has increased significantly through projects such as Free Cataract Surgeries for A Million Poor Patients in China.[24] The cataract surgery rate (CSR) in the Shanghai area increased from 1,741 in 2006 to 4,822 in 2016.[25 26] However, the findings of our study demonstrate the important contribution of URE to avoidable visual impairment in elderly Chinese aside from cataract and should raise awareness of the URE burden in China. An appropriate and cost-effective intervention for URE in older population should be considered during national health policy-making.

Strengths and limitations of the study

The strengths of the present study include a high response rate in a large

For peer review only - http://bmjopen.bmj.com/site/about/guidelines.xhtml

population-based sample, and standardized protocols based on the typical definition of URE. However, this study has several limitations. First, refractive error that impairs near vision, that is, presbyopia, was not assessed. Second, the gross domestic product per capita in Shanghai has approached the level of developed countries. All the subjects in the present study have health insurance, and ophthalmic consultations are easily accessed in this metropolitan area. We did not ask the reasons why they remained uncorrected for those subjects with URE in the present study, considering that economic factors and ophthalmic services should no longer be barriers. Further studies are needed to assess these issues. Furthermore, the present study was conducted in an urban population, which may limit the generalisability of the study findings. These results might not be extended to rural Chinese populations due to significant differences in social and economic factors between the urban and rural areas in mainland China.

Conclusions

In summary, this study suggests that URE is highly prevalent among the elderly Chinese population in urban China. These data provide a novel insight into the public health strategy for the Vision 2020 initiative to prevent avoidable blindness and visual impairment in the world's most populous nation. Further investigation is needed to identify the effects of URE on the quality of life of the elderly and the underlying reasons why they remain uncorrected.

For peer review only

For peer review only - http://bmjopen.bmj.com/site/about/guidelines.xhtml

Acknowledgments The authors thanks Jian Zhang (Zhongshan Ophthalmic Center, Sun Yat-sen University, Guangzhou, China) for providing suggestions on study sampling and statistical analysis.

Contributors HY and PZ conceived and designed the study; HY, YQ, XLiu, XC, WY, and XLi performed the study; HY, YQ, and Q.Z. analyzed the data; HY, YQ, and PZ wrote the paper. All the authors read and approved the submitted version of the manuscript.

Competing interests None declared.

Ethics approval Medical Ethics Committee of the Xinhua Hospital Affiliated to Shanghai Jiao Tong University School of Medicine (XHEC-C-2012-014).

Data sharing statement No additional data are available.

For peer review only

REFERENCES

1. Resnikoff S, Pascolini D, Mariotti SP, et al. Global magnitude of visual impairment caused by uncorrected refractive errors in 2004. *Bull World Health Organ* 2008;86(1):63-70.

2. Smith TS, Frick KD, Holden BA, et al. Potential lost productivity resulting from the global burden of uncorrected refractive error. *Bull World Health Organ* 2009;87(6):431-7.

3. Dandona R, Dandona L. Refractive error blindness. *Bull World Health Organ* 2001;79(3):237-43.

4. Owsley C, McGwin G, Jr., Scilley K, et al. Effect of refractive error correction

For peer review only - http://bmjopen.bmj.com/site/about/guidelines.xhtml

on health-related quality of life and depression in older nursing home residents. *Arch Ophthalmol* 2007;125(11):1471-7. doi: 10.1001/archopht.125.11.1471

5. Coleman AL, Yu F, Keeler E, et al. Treatment of uncorrected refractive error improves vision-specific quality of life. *J Am Geriatr Soc* 2006;54(6):883-90. doi: 10.1111/j.1532-5415.2006.00817.x
6. Schneider J, Leeder SR, Gopinath B, et al. Frequency, course, and impact of correctable visual impairment (uncorrected refractive error). *Surv Ophthalmol* 2010;55(6):539-60. doi: 10.1016/j.survophthal.2010.02.004
7. McCarty CA. Uncorrected refractive error. *Br J Ophthalmol* 2006;90(5):521-2. doi: 10.1136/bjo.2006.090233
8. Tabulation on the 2010 Population Census of the People's Republic of China. Available at http://www.stats.gov.cn/english/Statisticaldata/CensusData/rkpc2010/indexch.htm. Accessed December 6, 2017.
9. Huang S, Zheng Y, Foster PJ, et al. Prevalence and causes of visual impairment in Chinese adults in urban southern China. *Arch Ophthalmol* 2009;127(10):1362-7. doi: 10.1001/archophthalmol.2009.138
10. Zhu M, Tong X, Zhao R, et al. Visual impairment and spectacle coverage rate in Baoshan district, China: population-based study. *BMC Public Health* 2013;13:311. doi: 10.1186/1471-2458-13-311
11. Ye H, Zhang Q, Liu X, et al. Prevalence and associations of epiretinal

For peer review only

For peer review only - http://bmjopen.bmj.com/site/about/guidelines.xhtml

membrane in an elderly urban Chinese population in China: the Jiangning Eye Study. *Br J Ophthalmol* 2015;99(12):1594-7. doi: 10.1136/bjophthalmol-2015-307050

12. Ye H, Zhang Q, Liu X, et al. Prevalence of age-related macular degeneration in an elderly urban chinese population in China: the Jiangning Eye Study. *Invest Ophthalmol Vis Sci* 2014;55(10):6374-80. doi: 10.1167/iovs.14-14899

13. Rosman M, Wong TY, Tay WT, et al. Prevalence and risk factors of undercorrected refractive errors among Singaporean Malay adults: the Singapore Malay Eye Study. *Invest Ophthalmol Vis Sci* 2009;50(8):3621-8. doi: 10.1167/iovs.08-2788

14. Saw SM, Foster PJ, Gazzard G, et al. Undercorrected refractive error in Singaporean Chinese adults: the Tanjong Pagar survey. *Ophthalmology* 2004;111(12):2168-74. doi: 10.1016/j.ophtha.2004.05.032

15. Olshansky SJ, Carnes BA, Cassel CK. The aging of the human species. *Sci Am* 1993;268(4):46-52.

16. Attebo K, Ivers RQ, Mitchell P. Refractive errors in an older population: the Blue Mountains Eye Study. *Ophthalmology* 1999;106(6):1066-72. doi: 10.1016/S0161-6420(99)90251-8

17. Tielsch JM, Sommer A, Witt K, et al. Blindness and visual impairment in an American urban population. The Baltimore Eye Survey. *Arch Ophthalmol* 1990;108(2):286-90.

For peer review only - http://bmjopen.bmj.com/site/about/guidelines.xhtml

18. Schwab L, Steinkuller PG. Visual disability and blindness secondary to refractive errors in Africa. *Soc Sci Med* 1983;17(22):1751-4.

19. Kuang TM, Tsai SY, Hsu WM, et al. Correctable visual impairment in an elderly Chinese population in Taiwan: the Shihpai Eye Study. *Invest Ophthalmol Vis Sci* 2007;48(3):1032-7. doi: 10.1167/iovs.06-0616

20. Michon JJ, Lau J, Chan WS, et al. Prevalence of visual impairment, blindness, and cataract surgery in the Hong Kong elderly. *Br J Ophthalmol* 2002;86(2):133-9.

21. Foran S, Rose K, Wang JJ, et al. Correctable visual impairment in an older population: the blue mountains eye study. *Am J Ophthalmol* 2002;134(5):712-9.

22. Dimitrov PN, Mukesh BN, McCarty CA, et al. Five-year incidence of bilateral cause-specific visual impairment in the Melbourne Visual Impairment Project. *Invest Ophthalmol Vis Sci* 2003;44(12):5075-81.

23. Varma R, Wang MY, Ying-Lai M, et al. The prevalence and risk indicators of uncorrected refractive error and unmet refractive need in Latinos: the Los AngelesLatino Eye Study. *Invest Ophthalmol Vis Sci* 2008;49(12):5264-73. doi: 10.1167/iovs.08-1814

24. Lin H, Lin D, Long E, et al. Patient participation in free cataract surgery: a cross-sectional study of the low-income elderly in urban China. *BMJ Open* 2016;6(4):e011061. doi: 10.1136/bmjopen-2016-011061

25. Zhu M, Zhu J, Lu L, et al. Four-year analysis of cataract surgery rates in

For peer review only - http://bmjopen.bmj.com/site/about/guidelines.xhtml

Shanghai, China: a retrospective cross-sectional study. *BMC Ophthalmol* 2014;14:3. doi: 10.1186/1471-2415-14-3

26. THE LASTEST: Ranking of Chinese cataract surgery reporting situation in 2016. Available at: http://www.moheyes.com/(S(iwltcfbybpjl1x45sww14wjm))/News/Details/f5334a53-dd23-4bbe-a959-96c542b7262c. Accessed December 6, 2017.

For peer review only

Table 1. Prevalence rates of uncorrected refractive error by Gender and Age in the Jiangning Eye Study.

	All			Spectacle Wearers			Non-spectacle Wearers		
**Age Group (yrs.)**	***N***	**n**	**Prevalence Rate (%)**	***N***	**n**	**Prevalence Rate (%)**	***N***	**n**	**Prevalence Rate (%)**
Men									
50-59	297	35	11.8	81	8	9.9	216	27	12.5
60-69	321	47	14.6	120	14	11.7	201	33	16.4
70-79	158	46	29.1	48	11	22.9	110	35	31.8
80-95	99	41	41.4	28	7	25.0	71	34	47.9
Total population	875	169	19.3	277	40	14.4	598	129	21.6
Age-standardized prevalence (%)†			17.0 (14.5, 19.5)			13.3 (9.3, 17.3)			18.7 (15.6, 21.8)
P value for trend*			*P<0.001*			*P=0.013*			*P<0.001*
Women									
50-59	460	82	17.8	115	18	15.7	345	64	18.6
60-69	351	53	15.1	128	20	15.6	223	33	14.8
70-79	194	59	30.4	50	14	28.0	144	45	31.3
80-95	119	39	32.8	19	5	26.3	100	34	34.0
Total population	1124	233	20.7	312	57	18.3	812	176	21.7
Age-standardized prevalence (%)†			20.3 (17.9, 22.6)			18.5 (14.2, 22.9)			20.8 (18.0, 23.6)
P value for trend*			*P<0.001*			*P=0.079*			*P<0.001*
Both genders									
50-59	757	117	15.5	196	26	13.3	561	91	16.2
60-69	672	100	14.9	248	34	13.7	424	66	15.6
70-79	352	105	29.8	98	25	25.5	254	80	31.5
80-95	218	80	36.7	47	12	25.5	171	68	39.8
Total population	1999	402	20.1	589	97	16.5	1410	305	21.6
Age-standardized prevalence (%)†			18.7 (17.0, 20.4)			15.9 (13.0, 18.9)			19.7 (17.7, 21.8)
P value for trend*			*P<0.001*			*P=0.004*			*P<0.001*

**P* value for test of trend for age.

†Estimated prevalence (95% confidence interval) for projection by age-standardized to 2010 Chinese population census.

For peer review only

Table 2. Logistic Regression Model of the Predictors of Uncorrected Refractive Error.

**Variable**	***N***	**n (%)**	**Age OR (95% CI) ***	***P***	**Multivariate OR (95% CI) †**	***P***
**Age (per 1 year)**	1999	402 (20.1)	1.05 (1.04-1.06)	**<0.001**	1.04 (1.03-1.05)	**<0.001**
**Gender**				0.295		0.675
Male	875	169 (19.3)	1.0		1.0	
Female	1124	233 (20.7)	1.13 (0.90-1.41)		1.05 (0.84-1.32)	
**Education**				**0.008**		**0.019**
Secondary school and lower	902	217 (24.1)	1.36 (1.09-1.70)		1.34 (1.07-1.69)	
High school and above	1097	185 (16.9)	1.0		1.0	
**Income**				0.050		0.156
<5000	1327	292 (21.5)	1.28 (1.00-1.64)		1.20 (0.93-1.55)	
5000 and above	672	110 (16.4)	1.0		1.0	
**History of ocular diseases**				**0.007**		**0.009**
No	1500	315 (21.0)	1.0		1.0	
Yes	499	87 (17.4)	0.71 (0.55-0.91)		0.71 (0.55-0.92)	

OR = odds ratio; CI = confidence interval.

*Adjusted for age; †adjusted for age, gender, education, income, and history of ocular diseases.

For peer review only

For peer review only - http://bmjopen.bmj.com/site/about/guidelines.xhtml

Table 3. Prevalence rates and risk factors of uncorrected refractive error among Chinese population from the Jiangning and Other Eye Studies.

Study (Year of Study)	Country/ Region	*N*	Age (y)	URE in Study Population (%)	Definitions (VA: visual acuity)	Multivariate Risk Factors
Tanjong Pagar Survey[14] (1997-1998)	Singapore	1152	40–79	21.7%	BCVA - PVA ≥ 2 lines in the better eye of < 20/40	Older age, fewer years of education, not wearing spectacles, cataracts
Hong Kong Study[20] (1998)	Hong Kong	3441	60+	13.4%	Improving with pinhole to better than 20/60	NA
Shihpai Eye Study[19] (1999-2000)	Taiwan	1361	65+	9.6%	Improving to better than 20/40 on refraction	Older age, nonemmetropic eye, not wearing spectacles, lower level of education
Liwan Eye Study[9] (2003-2004)	Guangzhou	1399	50+	7.0%	Improvement to 20/40 or better with automated refraction	NA
Baoshan Study[10] (2009)	Shanghai	4545	60+	24.8%	Improvement of two or more lines in VA in the better eye after refraction	NA
Jiangning Eye Study (2012-2013) (current)	Shanghai	1999	50+	20.1%	BCVA - PVA ≥ 2 lines in the better eye of < 20/40	Older age, secondary school and lower of education, no history of ocular diseases

BCVA = best-corrected visual acuity; PVA = presenting visual acuity; NA = not applicable

For peer review only

For peer review only - http://bmjopen.bmj.com/site/about/guidelines.xhtml

STROBE 2007 (v4) Statement—Checklist of items that should be included in reports of *cross-sectional studies*

Section/Topic	Item #	Recommendation	Reported on page #
**Title and abstract**	1	(*a*) Indicate the study's design with a commonly used term in the title or the abstract	Page 1, 2
		(*b*) Provide in the abstract an informative and balanced summary of what was done and what was found	Page 2
**Introduction**			
Background/rationale	2	Explain the scientific background and rationale for the investigation being reported	Page 3, 4
Objectives	3	State specific objectives, including any prespecified hypotheses	Page 4
**Methods**			
Study design	4	Present key elements of study design early in the paper	Page 4
Setting	5	Describe the setting, locations, and relevant dates, including periods of recruitment, exposure, follow-up, and data collection	Page 4
Participants	6	(*a*) Give the eligibility criteria, and the sources and methods of selection of participants	Page 4
Variables	7	Clearly define all outcomes, exposures, predictors, potential confounders, and effect modifiers. Give diagnostic criteria, if applicable	Page 4
Data sources/ measurement	8*	For each variable of interest, give sources of data and details of methods of assessment (measurement). Describe comparability of assessment methods if there is more than one group	Page 5
Bias	9	Describe any efforts to address potential sources of bias	*NA*
Study size	10	Explain how the study size was arrived at	Page 4
Quantitative variables	11	Explain how quantitative variables were handled in the analyses. If applicable, describe which groupings were chosen and why	NA
Statistical methods	12	(*a*) Describe all statistical methods, including those used to control for confounding	Page 6
		(*b*) Describe any methods used to examine subgroups and interactions	NA
		(*c*) Explain how missing data were addressed	NA
		(*d*) If applicable, describe analytical methods taking account of sampling strategy	Page 4
		(*e*) Describe any sensitivity analyses	Page 6
**Results**			

For peer review only

Participants	13*	(a) Report numbers of individuals at each stage of study—eg numbers potentially eligible, examined for eligibility, confirmed eligible, included in the study, completing follow-up, and analysed	Page 6
		(b) Give reasons for non-participation at each stage	NA
		(c) Consider use of a flow diagram	NA
Descriptive data	14*	(a) Give characteristics of study participants (eg demographic, clinical, social) and information on exposures and potential confounders	Page 6
		(b) Indicate number of participants with missing data for each variable of interest	Page 6
Outcome data	15*	Report numbers of outcome events or summary measures	Page 6
Main results	16	(*a*) Give unadjusted estimates and, if applicable, confounder-adjusted estimates and their precision (eg, 95% confidence interval). Make clear which confounders were adjusted for and why they were included	Page 7
		(*b*) Report category boundaries when continuous variables were categorized	Page 7
		(*c*) If relevant, consider translating estimates of relative risk into absolute risk for a meaningful time period	NA
Other analyses	17	Report other analyses done—eg analyses of subgroups and interactions, and sensitivity analyses	Page 7
**Discussion**			
Key results	18	Summarise key results with reference to study objectives	Page 8
Limitations	19	Discuss limitations of the study, taking into account sources of potential bias or imprecision. Discuss both direction and magnitude of any potential bias	Page 10, 11
Interpretation	20	Give a cautious overall interpretation of results considering objectives, limitations, multiplicity of analyses, results from similar studies, and other relevant evidence	Page 8-10
Generalisability	21	Discuss the generalisability (external validity) of the study results	Page 10
**Other information**			
Funding	22	Give the source of funding and the role of the funders for the present study and, if applicable, for the original study on which the present article is based	NA

*Give information separately for cases and controls in case-control studies and, if applicable, for exposed and unexposed groups in cohort and cross-sectional studies.

Note: An Explanation and Elaboration article discusses each checklist item and gives methodological background and published examples of transparent reporting. The STROBE checklist is best used in conjunction with this article (freely available on the Web sites of PLoS Medicine at http://www.plosmedicine.org/, Annals of Internal Medicine at http://www.annals.org/, and Epidemiology at http://www.epidem.com/). Information on the STROBE Initiative is available at www.strobe-statement.org.

For peer review only
